# Draft Genome Sequence of *Streptomyces albulus* Strain CCRC 11814, an ε-Poly-l-Lysine-Producing Actinomycete

**DOI:** 10.1128/genomeA.00696-13

**Published:** 2013-09-05

**Authors:** Amanda Dodd, Dirk Swanevelder, Jonathan Featherston, Karl Rumbold

**Affiliations:** School of Molecular and Cell Biology, University of the Witwatersrand, Johannesburg, South Africaa; Biotechnology Platform, Agricultural Research Council, Onderstepoort, South Africab

## Abstract

Here, we report the draft genome sequence of *Streptomyces albulus* strain CCRC 11814, a soil-dwelling, Gram-positive bacterium. *S. albulus* produces ε-poly-l-lysine, which has diverse antimicrobial activity. The genome is 9.43 Mb in size, with a G+C content of 72.2%, and contains 9,177 protein-coding sequences.

## GENOME ANNOUNCEMENT

Members of the genus *Streptomyces*, the largest genus of actinomycetes, are able to produce an extensive scope of secondary metabolites that are bioactive in a wide range of applications. In the pharmaceutical industry, they produce >67% of naturally derived antibiotics, as well as compounds such as antitumor agents, immunosuppressants, and antifungals (1, 2). The *Streptomyces*-produced bioactive molecules market is over $30 billion per year (3).

*Streptomyces albulus* produces a valuable secondary metabolite, ε-poly-l-lysine (PL) (4). PL, a homopolymer consisting of 25 to 30 lysine residues, is active against various microorganisms, including Gram-positive and -negative bacteria, fungi, and phages (5, 6). This antimicrobial activity and its water solubility make it ideal for use as a food preservative.

*S. albulus* CCRC 11814 was kindly donated by Yoshimitsu Hamano from the Fukui Prefectural University, Japan. Genomic DNA was isolated from a liquid culture using the ZR fungal/bacterial DNA MiniPrep kit (Zymo Research). Genomic DNA paired-end libraries were generated with the NextEra DNA sample preparation kit (Illumina) and indexed using the NextEra index kit (Illumina). Paired-end (2 × 250 bp) sequencing was performed on a MiSeq (Illumina) using the MiSeq reagent kit v2 at the Agricultural Research Council (ARC) Biotechnology Platform. Demultiplexing and quality and adapter trimming were performed with Casava 1.7. A total of 7,866,954 paired-end reads at 174 × coverage were obtained from this workflow. The genome was assembled using the *de novo* assembly tool in the CLC Genomics Workbench v6 (CLC bio). This assembly produced 242 contigs with an average length of 39,836 bp and an N_50_ of 79,110 bp. The *S. albulus* genome is 9.43 Mb in size, with a high G+C content (72.2%) that is similar to that of other *Streptomyces* species. Genome annotation was performed using the NCBI Prokaryotic Genome Automatic Annotation Pipeline (PGAAP), resulting in 9,177 protein-coding sequences (CDS) being identified. The genome also contains 69 tRNA genes (determined by tRNAscan-SE) and 4 rRNA genes with 1 rRNA operon (16S, 23S, and 5S) located on contig 198 (determined by RNAmmer).

In *S. albulus*, the amino acid biosynthetic pathway produces l-lysine, which is the precursor for PL production. The initial reaction of amino acid biosynthesis, catalyzed by aspartate kinase, is under feedback regulation by its products. *S. albulus* is unique in that it does not suffer from this feedback regulation of aspartate kinase and can produce l-lysine, and therefore PL, in high quantities (4). The ultimate step in PL biosynthesis is the polymerization of l-lysine, a reaction catalyzed by ε-poly-l-lysine synthetase. This draft genome sequence will allow for the investigation of these genes and other genes in more detail, thereby allowing us to identify the target genes for metabolic engineering, especially when adapting the strain for industrial bioprocessing and second-generation feedstock utilization (7).

### Nucleotide sequence accession numbers.

This whole-genome shotgun project has been deposited at DDBJ/EMBL/GenBank under the accession no. AROY00000000. The version described in this paper is version AROY02000000.
